# Probiotic Supplementation for Rheumatoid Arthritis: A Promising Adjuvant Therapy in the Gut Microbiome Era

**DOI:** 10.3389/fphar.2021.711788

**Published:** 2021-07-23

**Authors:** Margarida Ferro, Sofia Charneca, Eduardo Dourado, Catarina Sousa Guerreiro, João Eurico Fonseca

**Affiliations:** ^1^ Laboratório de Nutrição, Faculdade de Medicina, Universidade de Lisboa, Lisboa, Portugal; ^2^ Serviço de Reumatologia e Doenças Ósseas Metabólicas, Centro Hospitalar Universitário Lisboa Norte, Centro Académico de Medicina de Lisboa (CAML), Lisboa, Portugal; ^3^ Unidade de Investigação em Reumatologia, Instituto de Medicina Molecular, Faculdade de Medicina, Universidade de Lisboa, CAML, Lisboa, Portugal; ^4^ Instituto de Saúde Ambiental, Faculdade de Medicina, Universidade de Lisboa, Lisboa, Portugal

**Keywords:** dysbiosis, inflammation, autoimmunity, rheumatology, fermented foods

## Abstract

Rheumatoid arthritis (RA) is a chronic immune-mediated inflammatory disease that ultimately leads to joint destruction and functional disability. Although the exact etiology of RA is not fully understood, it is well established that gut microbiota (GM) plays a vital role in the pathogenesis of RA, with accumulating evidence suggesting that gut dysbiosis induces a chronic inflammatory response that may be linked to disease development. Of interest, patients with RA have significant changes in the intestinal microbiota compared to healthy controls, and several studies have suggested the use of probiotics as a possible adjuvant therapy for RA. Benefits of probiotic supplementation were reported in animal models of arthritis and human studies, but the current evidence regarding the effect of probiotic supplementation in the management of RA remains insufficient to make definite recommendations. Several different strains of *Lactobacillus* and *Bifidobacteria*, as single species or in mixed culture, have been investigated, and some have demonstrated beneficial effects on disease activity in RA human subjects. As of now, *L.casei* probiotic bacteria seems to be the strongest candidate for application as adjuvant therapy for RA patients. In this review, we highlight the role of GM in the development and progression of RA and summarize the current knowledge on the use of probiotics as a potential adjuvant therapy for RA. We also review the proposed mechanisms whereby probiotics regulate inflammation. Finally, the role of fermented foods is discussed as a possible alternative to probiotic supplements since they have also been reported to have health benefits.

## Introduction

Rheumatoid Arthritis (RA) is a chronic immune-mediated inflammatory disorder that involves the synovial membranes of multiple joints (Sewell and Trentham, 1993; McInnes and Schett, 2017). The inflammatory process underlying this disease causes cartilage and bone destruction, damaging the joint structure and (Kang et al., 2017) leading to functional disability (Firestein, 2003; Smolen et al., 2016; Kalinkovich et al., 2018). In addition, systemic inflammation may impact other organs and systems, such as the cardiovascular, pulmonary, skeletal bone, and brain (McInnes and Schett, 2011). RA is characterized by autoantibodies production in most patients, such as rheumatoid factor and anti-citrullinated protein antibodies (McInnes and Schett, 2011). Although the exact etiology of RA remains unknown, it has become evident that besides genetic factors, the environment (including the *internal* environment, the microbiome) plays a pivotal role in disease onset (Scherer et al., 2020).

RA patients have compositional and functional alterations in the gut microbiota (GM) (Zhang et al., 2015), and a significant decrease in microbial diversity compared with healthy controls has been reported (Chen et al., 2016). Moreover, the GM of RA patients exhibited decreased diversity with increased disease duration (Chen et al., 2016). *Faecalibacterium* is one of the most abundant *Firmicutes* in the human gut that produces butyrate, and a decreased abundance of *Faecalibacterium* and other butyrate producing taxa, such as *Flavobacterium*, have been reported in RA patients (Picchianti-Diamanti et al., 2018). On the other hand, the GM of RA patients has a significant increase in the order of *Lactobacillales* (Chen et al., 2016; Picchianti-Diamanti et al., 2018), and a higher variety of *lactobacilli* compared to healthy controls (Liu et al., 2013). Accordingly, an increase in the *Lactobacillaceae* family and the *Lactobacillus* genus in mice susceptible to collagen-induced arthritis (CIA) have been reported (Liu et al., 2016). Interestingly, the administration of some *Lactobacillus* species, seems to exert beneficial effects in RA clinical signs, which suggests that different *Lactobacilli* may have different roles in RA pathogenesis and disease activity modulation (Alipour et al., 2014; Vaghef-Mehrabany et al., 2014). In early RA patients, a significant increase of *Prevotella* genus has been frequently found in comparison to healthy controls, in particular *Prevotella copri* (*P. copri*) (Maeda et al., 2016; Paul et al., 2021; Reyes-Castillo et al., 2021). Given that the GM of RA patients differs from the general population and that anti-rheumatic drugs can exert positive effects on its regulation (Croia et al., 2019), microbiome research in the field of Rheumatology is expanding significantly (Manasson et al., 2020). Mounting evidence supports the existence of a reciprocal connection between drugs and GM, which can influence each other and have an impact on therapeutic outcomes (Bhat et al., 2017). Specifically, methotrexate (MTX) was shown to modify GM composition, partly restoring the microbial balance altered by the disease (Zhang et al., 2015; Picchianti-Diamanti et al., 2018). Moreover, the partial restoration of a beneficial microbiota induced mainly by anti tumor necrosis factor (TNF) drugs (as shown for etercept) can contribute to the clinical efficacy of these agents. A deeper understanding of the alterations occurring in the GM of patients on different therapeutic regimens could help set up individualized and supportive therapeutic strategies providing patients with more effective and safe care (Picchianti-Diamanti et al., 2018). In line with this, GM modulation and its interactions with the host have been reported as a strategy to prevent and control rheumatic diseases (Van De Wiele et al., 2016).

Probiotics are defined as *live microorganisms that, when administered in adequate amounts, confer a health benefit on the host* (Hill et al., 2014). Several studies have suggested the use of probiotics as a possible adjuvant therapy for RA patients (Ciccia et al., 2016; Wang et al., 2016; Reyes-Castillo et al., 2021). Various mechanisms whereby probiotics affect RA have been proposed, but are still poorly scientifically supported. Currently, most of the available research on this topic was conducted in animal models of arthritis. Exogenous bacteria can have a transient and subject-specific effect on the GM and, by its modification, can improve dysbiosis-related diseases (Zhang et al., 2016), such as RA. Since the probiotic effect is strain-dependent (Butel, 2014), the most appropriate strain must be chosen.

RA is a major global public health challenge with increasing age-standardized prevalence and incidence (Safiri et al., 2019). Despite the substantial advances with novel pharmacological therapies, the impact of RA on patient’s functional capacity and quality of life remains a significant issue. Most patients have a chronic persistent form of the disease, as full remission is uncommon and sustained remission is even more unlikely to occur. Moreover, in order to control this disease, chronic treatment is needed, and multiple drug adverse effects often accumulate over the years. Indeed, there are still considerable unmet needs in RA management, and new safe treatment approaches that complement the existing ones are required (Smolen et al., 2016).

This paper aims to provide an up-to-date review of both animal and human studies investigating the effects of probiotics in RA and the proposed mechanisms whereby probiotics regulate inflammation. Since fermented foods can be used as a probiotic carrier and contain health-promoting components (Melini et al., 2019), we address their potential use in this context as a possible alternative to probiotic supplements.

## Gut Microbiota, Immune System and Rheumatoid Arthritis

It is now well established that more than 100 trillion microorganisms, primarily bacteria, colonize the human oral-gastrointestinal tract, most residing in the distal intestine (Kamada et al., 2013). In recent years, there has been a dramatic increase in the interest regarding the composition and function of GM, resulting in a large body of evidence supporting GM as a crucial component in shaping host physiology and maintaining gut and immune homeostasis (Derrien and van Hylckama Vlieg, 2015).

The clinical picture of RA results from a complex interaction between various factors, including autoantibodies and signal transduction pathways of the innate and adaptive immune system (Croia et al., 2019). In RA patients, joint tissue is typically infiltrated by immune cells such as T cells, B cells, and macrophages, producing a variety of pro-inflammatory cytokines facilitating inflammation and eventually leading to tissue destruction (Volkov et al., 2020). Throughout life, GM plays a fundamental role in the induction, education, and function of the immune system, as well as the individuals’ response to self-antigens (Belkaid and Hand, 2014; D’Amelio and Sassi, 2018; Wu and Wu, 2012). The modulation of GM may regulate the mechanism of gut immune tolerance, as it influences the number and function of colonic regulatory T cells (Tregs) (Tanoue et al., 2016). Tregs suppress inappropriate activation of effector T cells by secreting anti-inflammatory cytokines (Kalinkovich and Livshits, 2019; Kayama et al., 2020). On the other hand, the mucosal immune system has a crucial role in developing and maintaining a healthy GM (X. Wu et al., 2016). Due to this interdependent relationship, gut dysbiosis, a compositional and functional alteration of GM (Levy et al., 2017), may influence host susceptibility to many immune-mediated diseases such as RA, type 1 diabetes, multiple sclerosis, and systemic lupus erythematosus (de Oliveira et al., 2017). Additionally, there is emerging literature reporting on the role of changes in GM in the pathogenesis of chronic immune-mediated inflammatory disorders, including RA (Ciccia et al., 2016; Kalinkovich et al., 2018; Zhong et al., 2018). Deregulation of host responses as a consequence of gut dysbiosis could affect distant anatomical sites through the activation of host immune responses, and this could be the mechanism contributing to the onset of an idiopathic inflammatory condition like RA (Cho and Blaser, 2012).

**FIGURE 1:**
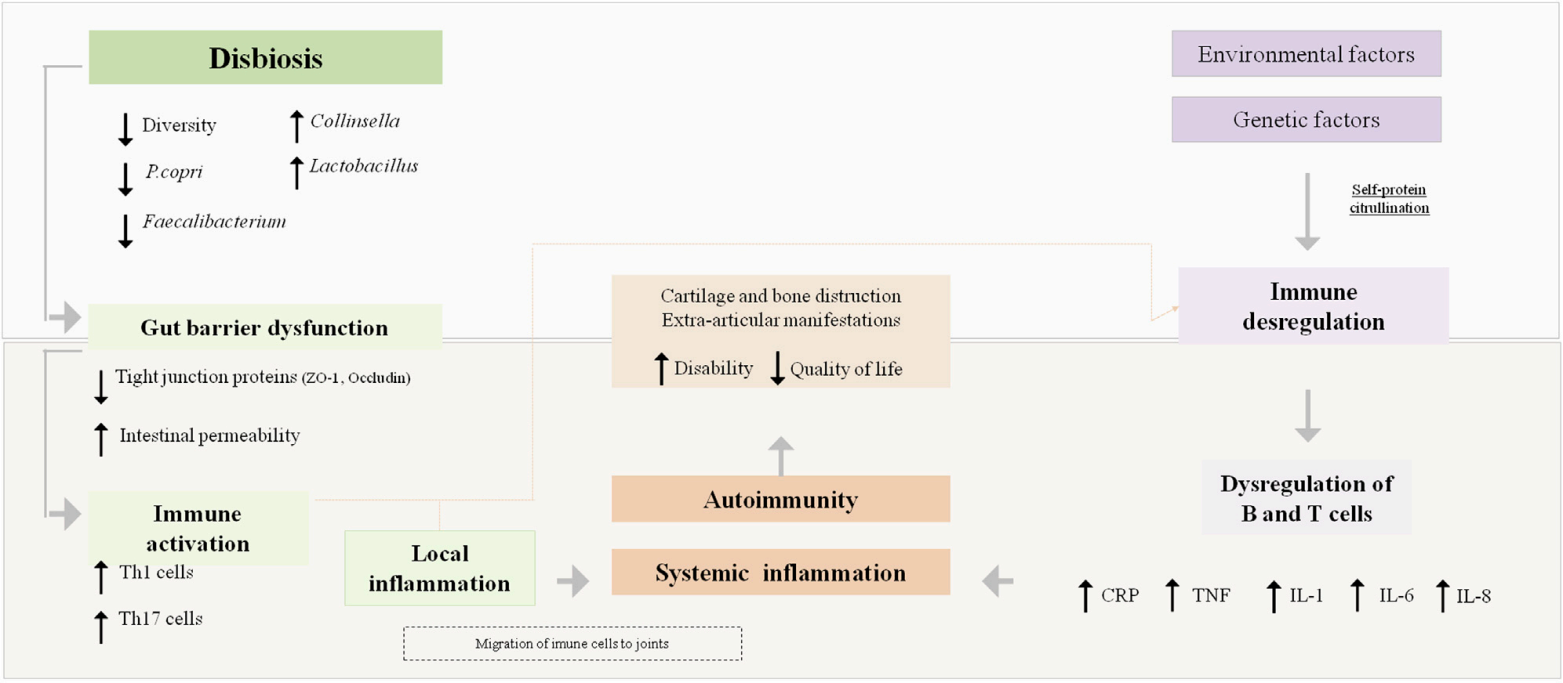
Rheumatoid arthritis pathophysiology and the proposed mechanisms by which gut microbiota could influence its pathogenesis. CRP, C-reactive; P. Copri, Prevotella copri; ZO-1, Zonula occluclens-1; IL, Interleukin; TNF, tumour necrosis factor.

To support the hypothesis that changes in GM composition play a significant role in the onset and progression of RA (Horta-Baas et al., 2017; Kang et al., 2017), several mechanisms by which GM is associated with arthritis have been proposed. These include regulating the host’s immune system (triggering T cell differentiation), activating antigen-presenting cells (APCs) through an effect on Toll-like receptors (TLRs) or NOD-like receptors (NLRs), promoting the citrullination of peptides by enzymatic action, antigenic mimicry, and increasing the intestinal mucosal permeability (Horta-Baas et al., 2017; Guerreiro et al., 2018). Regarding the effect on the expression of TLRs of APCs, this may contribute to an imbalance in the Th17/Treg cell ratio and this local immune response could lead to systemic autoimmunity (Horta-Baas et al., 2017). Thus, the existing literature suggests that GM could contribute to or prevent the expansion of autoimmunity and inflammation during the preclinical and clinical phases of RA, and GM could influence transitions between these phases (Wilson et al., 2020). Figure 1 summarizes the pathophysiology of RA and the proposed mechanisms whereby GM could participate in triggering autoimmunity and systemic inflammation in susceptible individuals.

Since the diet is an essential environmental factor impacting intestinal microbiota composition, increasing attention has been given to its role in the pathogenesis, progression, and activity of rheumatic diseases (Gioia et al., 2020). In this regard, the use of probiotic bacteria has been suggested as a possible strategy to correct gut dysbiosis and promote the homeostasis of a healthy microbiota, having an impact on systemic immune responses and thus could be used as adjuvant therapy to treat immune-mediated diseases (Gareau et al., 2010; de Oliveira et al., 2017).

## Mechanisms Underlying Probiotics Effects on Gut Microbiota, Immune System and Rheumatoid Arthritis

A link between the composition and activity of GM and human health and disease has been previously described (Azad, Sarker, Li, et al., 2018). Although the local effects of probiotic bacteria on gut health are well reported, the mechanisms behind their systemic anti-inflammatory and immunomodulating potential have not been wholly explored (Vieira et al., 2016; Plaza-Diaz et al., 2019; Oliviero and Spinella, 2020). A set of mechanisms whereby probiotics regulate inflammation have been postulated, which can be exerted not only via direct immune system modulation, but also through indirect mechanisms (La Fata et al., 2018; Kalinkovich and Livshits, 2019).

### Probiotic’s Direct Mechanisms of Immune System Modulation

Specific probiotic bacteria modulate the immune response by affecting different cells involved in innate and acquired immunity, such as epithelial cells and dendritic cells (DCs), natural killer cells (NK), macrophages, and lymphocytes (Bermudez-Brito et al., 2012; La Fata et al., 2018; Cristofori et al., 2021).

The innate immune system develops the primary response to pathogens after activation of the pattern recognition receptors (PRRs), which are expressed on immune and non-immune cells, such as NK cells, DCs, macrophages, fibroblasts, and epithelial cells (Bermudez-Brito et al., 2012; Plaza-Diaz et al., 2019; Cristofori et al., 2021). Toll-like receptors (TLRs) are the most widely studied PRRs, which can activate signaling pathways that affect cell proliferation and cytokine production to modulate the immune system (Ferlazzo et al., 2011; Cristofori et al., 2021). It is well established that probiotics can downregulate TLR expression, reducing inflammation (Gómez-Llorente et al., 2010).

Concerning the adaptive immune response, T cells are central to immune balance (Plaza-Diaz et al., 2019). Inflammatory responses driven by T helper (Th)1 and Th17 cells protect the host from pathogens, but their overactivation is linked to the pathogenesis of detrimental inflammation. The adaptive immune cells Foxp3+ Tregs suppress inappropriate activation of effector Th cells by secreting anti-inflammatory cytokines, such as IL-10, modulating the immune response (Kalinkovich and Livshits, 2019; Peters et al., 2019; Kayama et al., 2020). Probiotics have also been reported to influence cytokine production by APCs, which initiates adaptive responses (Azad, Sarker, and Wan, 2018). Beyond the described immunomodulatory properties involving DCs and T cells, some probiotic strains also have a role in increasing the production of secretory IgA once they promote the differentiation of B cells into plasma cells (Liu, Tran, et al., 2018). Secretory IgA provides a defense against pathogens by limiting bacterial adhesion to the epithelium and preventing the penetration of host tissue (Azad, Sarker, and Wan, 2018; Liu, Tran, et al., 2018).

### Probiotic’s Indirect Mechanisms of Immune System Modulation

Probiotics can also interact with the host immune system through indirect mechanisms, which involve the modulation of GM. The mechanisms by which probiotic strains have been proposed to modulate GM include regulating the gut epithelial barrier and the mucus layer characteristics, secretion of antimicrobial compounds and competition with pathogenic bacteria (Vieira et al., 2016; Jethwa and Abraham, 2017; Cristofori et al., 2021).

The gut epithelium, which separates the luminal environment from the intestinal milieu, has a key role in assuring the permeability to nutrients and other molecules, as well as blocking the entry of toxins and other microorganisms (Deane et al., 2017; Van Spaendonk et al., 2017). Tight Junction (TJ) proteins, located in the apical part of the intestinal epithelial cells, are crucial elements to ensure the functionality and integrity of the mucosal barrier (Ulluwishewa et al., 2011; Lee, 2015). When an alteration in the expression or localization of TJ proteins occurs, the epithelial barrier function is compromised due to increased permeability (Ulluwishewa et al., 2011). The use of probiotics and the consequent increase in the short-chain fatty acids (SCFA) release, particularly butyrate, has been reported to enforce the gut barrier function as butyrate strengthens the barrier through increased expression of TJ components zonula occludens (ZO)-1, ZO-2, and cingulin (Bordin et al., 2004; Deane et al., 2017; Liu, Tran, et al., 2018).

The intestinal epithelium is covered by a viscoelastic mucus layer, mainly composed of mucins, high-molecular-weight glycoproteins produced by goblet cells (La Fata et al., 2018). Mucins are responsible for building a protective barrier containing digestive enzymes, promoting food passage, and at the same time prevent the entry of bacteria into the lamina propria by blocking their adhesion to the epithelial cells (Corfield et al., 2000; Derrien et al., 2010; De Santis et al., 2015). The intestinal mucus layer has a primary role in protecting against mechanical, chemical, and biological attacks to the gut and contributes to the maintenance of intestinal homeostasis (Paone and Cani, 2020). Some probiotic strains have been reported to regulate mucin expression, altering the properties of the mucus layer and indirectly regulating the gut immune system (La Fata et al., 2018). Examples include the adherent *Lactobacillus spp*, which can stimulate MUC3 expression in human intestinal epithelial cells and MUC2 production and secretion (Sicard et al., 2017; Bron et al., 2017), and *Lactobacillus reuteri* (*L. reuteri*), which has a protective effect against dextran sulfate sodium-induced colitis in mice, increasing the mucus layer thickness (Ahl et al., 2016).

Specific probiotic-modulated local and systemic metabolites have been reported to have anti-inflammatory and antimicrobial functions, such as SCFA, dietary tryptophan, adenosine, and histamine (Liu, Alookaran, et al., 2018). One of the primary mechanisms by which probiotics compete in this environment is through competitive exclusion, by which they adhere to the intestinal mucosa and prevent the subsequent entry of pathogens into the lamina propria (Liu, Tran, et al., 2018; Santis et al., 2015). Moreover, the adhesion of probiotic microorganisms to epithelial cells may trigger a signaling cascade, leading to immunological modulation (Markowiak and Sli˙, 2017). As previously described, SCFAs exert an indirect anti-inflammatory effect through improving intestinal barrier function (Bodkhe et al., 2019; Kolodziejczyk et al., 2019). Butyrate is particularly relevant in modulating inflammation, as it inhibits histone deacetylase and regulates the expression of numerous pro-inflammatory genes, inducing the differentiation and expansion of Tregs and regulating cytokine production (Koh et al., 2016; Liu et al., 2021; Liu, Tran, et al., 2018; Peters et al., 2019).

### Probiotic’s Mechanisms and the Pathophysiology of Rheumatoid Arthritis

Crosstalk between gut epithelium, immune system, and commensal bacteria is key to starting the systemic inflammatory response (Y. Liu, Tran, et al., 2018). An imbalance between anti-inflammatory and pro-inflammatory cytokines, including interleukin (IL)-1β, TNF, interferon (IFN)-γ, IL-6, IL-12, and IL-17, plays a central role in the inflammatory processes involved in the pathogenesis of RA (Smolen and Steiner, 2003; So et al., 2008; Amdekar et al., 2011).

The proposed mechanism for the gut-joint axis in inflammatory arthritis is related to the hyperpermeability of the gut wall, which leads to the exposure of immune system to microorganisms, leading to a systemic immune response that triggers a local inflammatory process within the joints (Jethwa and Abraham, 2017; Liu et al., 2021).

Considering the perturbed GM as a pivotal trigger in the pathogenesis of RA, interest has emerged regarding the clinical interest of probiotics to correct gut dysbiosis and downregulate the pro-inflammatory cytokine cascade implicated in inflammatory arthritis (Wang et al., 2016; Mohammed et al., 2017; Lowe et al., 2020). Probiotics upregulate regulatory cytokines produced by Tregs or tolerogenic DCs in the gut. Cytokines travel to target organs and expand Tregs that traffic to inflammation sites (Marietta et al., 2016). Probiotic-driven metabolic products, such as SCFA, also impact immune response and systemic inflammation by regulating immune cell function (Oliviero and Spinella, 2020). SCFA as regulators of several leukocyte functions including production of eicosanoids and chemokines and cytokines, such as TNF, IL-2, IL-6 and IL-10, exhibit anti-inflammatory properties (Vinolo et al., 2011; Kalinkovich and Livshits, 2019). Moreover, probiotic bacteria and its metabolic products can keep a balance between tolerance to the intestinal microflora and resistance against harmful bacterial colonization, adherence, and translocation (Mohammed et al., 2017). These properties may be useful to correct the hyperpermeability of gut wall proposed for the gut-joint axis in inflammatory arthritis.

A meta-analysis of randomized trials investigating the effect of *Lactobacillus* as single species or in mixed cultures with *Bifidobacterium species* concluded that probiotic supplementation reduced serum levels of IL-6 (Mohammed et al., 2017). Another systematic review and meta-analysis that investigated the effectiveness of *Lactobacillus casei (L. casei)* supplementation in RA reported that a significant reduction of C-reactive protein (CRP) was achieved with this specific strain (Rudbane et al., 2018). Studies have also reported that *L. casei* might help alleviate RA symptoms and suppress pro-inflammatory cytokines in individuals undergoing treatment with disease-modifying anti-rheumatic drugs (DMARDs), which suggests a positive synergistic effect between DMARDs and probiotics on arthritis (Alipour et al., 2014; Pan et al., 2017, 2019).

**FIGURE 2:**
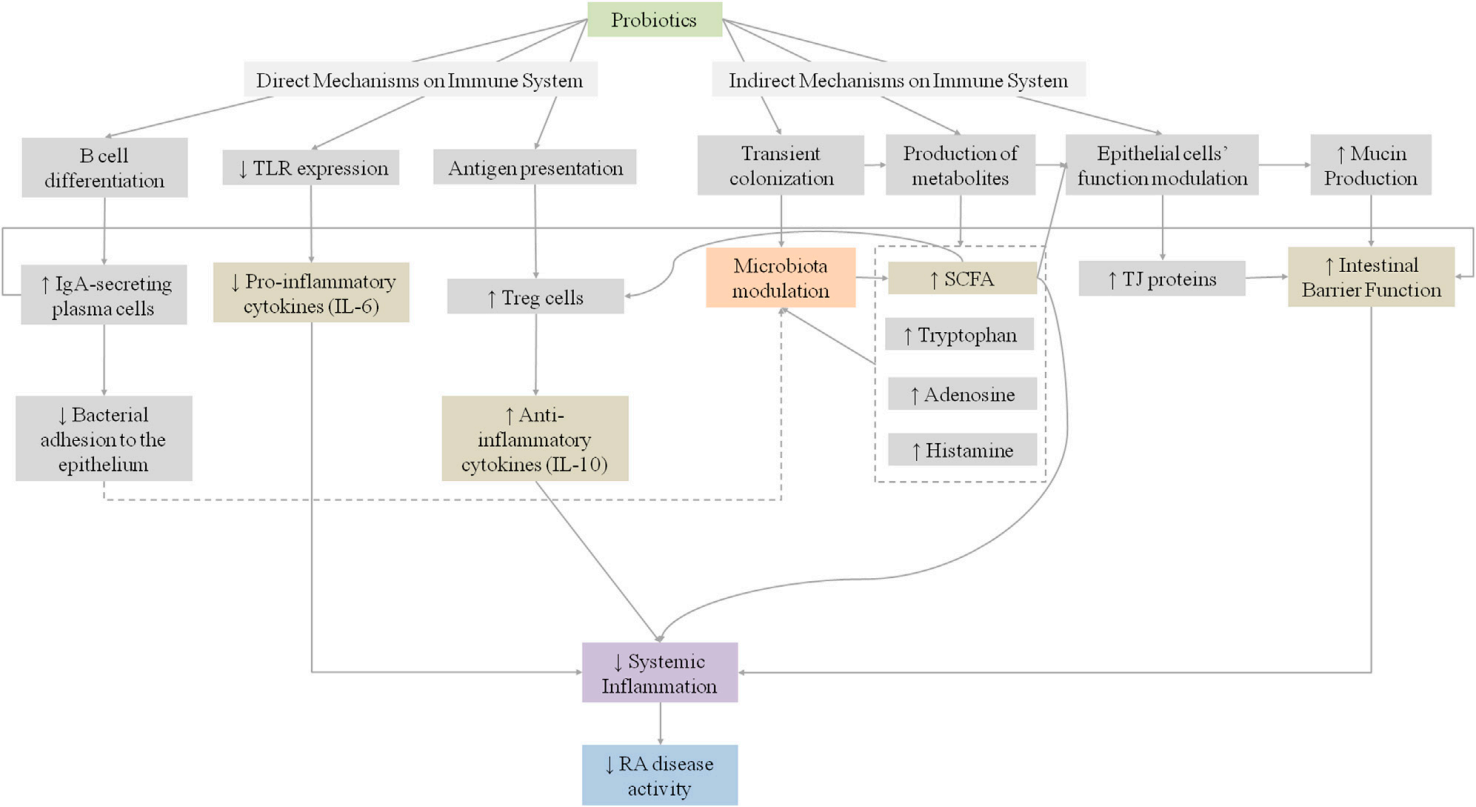
Proposed mechanisms for the influence of probiotics on systemic inflammation in rheumatoid arthritis patients. IgA, Immunoglobulin A; IL, Interleukin; RA, Rheumatoid; SCFA, short Chain Fatty Acids; TJ, Tight Junctions; TLR, Toll-like receptors.

These findings suggest that the administration of probiotic bacteria may have a beneficial effect on the inflammatory activity of RA, through the regulation of cytokine production, improvement of the intestinal barrier function, and its positive synergistic effect with DMARDs. Figure 2 summarizes the proposed mechanisms for the influence of probiotics on RA.

## Experimental Evidence of Probiotic Effects on Rheumatoid Arthritis Prevention and Treatment

### Experimental Evidence From Animal Models

This section will review the current evidence for microbiome manipulation by using probiotic bacteria in animal models of arthritis for both disease prevention and treatment.

#### Studies With the Collagen-Induced Arthritis Animal Model

Several studies have proposed a link between GM and CIA development with oral administration of several bacterial strains in mice.

A study conducted by Fan et al. compared the effects of a preventive and therapeutic treatment with *Bifidobacterium adolescentis* (*B. adolescentis*) in CIA rats (Fan et al., 2020). Preventive *B. adolescentis* administration had better results in reducing the clinical symptoms, rebalancing the pro- and anti-inflammatory responses, and reversing the gut dysbiosis than late *B. adolescentis* treatment. Early probiotic administration performed better in promoting SCFAs production, had significant higher Tregs cells frequency and lower levels of TNF compared to the late *B. adolescentis* treated group. Moreover, SCFA positively correlated with Tregs and negatively correlated with pro-inflammatory cytokines in the early treated group (Fan et al., 2020). These findings suggest that the introduction of *B. adolescentis* before arthritis can impact the onset of arthritic inflammation, and support that GM manipulating therapies should be provided at an early stage of the disease or even before disease occurrence.

Another study investigated the effects of the oral administration of *L. casei Shirota* (LcS) in a mouse model of CIA (Kato et al., 1998). LcS administration during induction of CIA suppressed the abnormal anti-type II collagen antibody production and delayed onset and reduced severity of CIA. It was concluded that oral administration of LcS reduced the humoral and cellular immune responses to CIA, which could result in reduced rates of CIA development in mice (Kato et al., 1998).

Yamashita et al. evaluated the effect of oral administration of *L. helveticus* SBT2171 in reducing the incidence and progression of CIA (Yamashita et al., 2017). Oral administration of *L. helveticus* SBT2171 significantly relieved joint swelling and suppressed weight loss. These findings suggested that *L. helveticus* SBT2171 can downregulate the abundance of immune cells and the production of anti-type II collagen antibodies and IL-6, suppressing CIA symptoms indicating its potential for use in the prevention of RA (Yamashita et al., 2017).

Amdekar et al. assessed the therapeutic efficacy of *L. casei* in a CIA model of arthritis and reported a therapeutic effect of this probiotic when administered after the onset of arthritis (Amdekar et al., 2011). There was a significant reduction in the arthritis score with a significantly decreased secretion of pro-inflammatory cytokines (TNF and IL-6) and an increased concentration of IL-10, an anti-inflammatory cytokine (Amdekar et al., 2011). The authors suggested that the exerted anti-inflammatory effect of *L. casei* was a result of Cyclooxygenase (COX)-2 and NF-κB inactivation (Amdekar et al., 2011). Previous research on the COX-2 have already sugested that it has a key role in inflammation in RA (Kang et al., 1996), as COX-2 has been pointed out as the responsible for the overproduction of prostaglandins. Prostaglandins are implicated in different phases of inflammatory reactions, and its synthesis is down regulated by anti-inflammatory cytokines, such as IL-10. In this regard, the proposed mechanism provided by Amdekar et al. for the obtained effect with L. casei in a CIA model, involves prostaglandins inhibition due to an increased secretion of IL-10 promoted by the probiotic treatment.

Another study from Amdekar et al. evaluated the anti-inflammatory and antioxidant properties of *L. casei* and *Lactobacillus acidophilus* (*L. acidophilus*) as a therapeutic protocol in an experimental model of CIA. The results suggested that *L. casei* and *L. acidophilus* exhibit antiarthritic and anti-inflammatory properties by suppressing IL-6, TNF, IL-17, and IL-1β production and upregulating IL-10 and IL-4 (Amdekar et al., 2013). Along with the reported imbalances between pro-inflammatory and anti-inflammatory cytokines, which have been reported to play an important role in initiation and pathogenesis of arthritis, prostaglandins, nitric oxide, and reactive oxygen species (ROS) are also released at the site of inflammation in many rheumatic diseases, damaging the cartilage and the components of extracellular matrix. In this study, *L. casei* and *L. acidophilus* significantly decreased lipid peroxidation and catalase (CAT) levels, and increased the concentration of glutathione peroxidase (GPx), glutathione (GSH) and superoxide dismutase (SOD) (Amdekar et al., 2013). These findings are particularly relevant as they suggest that the beneficial effects of *L. casei* and *L. acidophilus* are due, not only to their anti-inflammatory effect, but also to their antioxidant properties.

In a study conducted by Marietta et al., an isolated human gut commensal *Prevotella histicola* (*P. histicola*) was tested for treating CIA in HLA-DQ8 transgenic mice in prophylactic and therapeutic protocols (Marietta et al., 2016). Mice were monitored for the onset and progression of CIA. Treating mice with *P. histicola* significantly decreased the incidence and severity of arthritis compared to controls (Marietta et al., 2016). The microbial modulation of arthritis was dependent on the generation of Tregs in the gut, resulting in suppression of Th17 response and increased release of IL-10. Moreover, treatment with *P. histicola* improved intestinal barrier function by increasing the expression of TJ proteins, ZO-1 and occludin (Marietta et al., 2016).

So et al. investigated the effect of *L. casei* in suppressing the inflammatory immune responses of RA by testing its impact on the effector functions of CD4^+^ T cells (So et al., 2008). This study demonstrated that *L. casei* could effectively suppress RA-related pathways by simultaneously down-regulating Th1 effector functions and upregulating anti-inflammatory IL-10 (So et al., 2008). Additionally, oral administration of *L. casei* suppressed arthritis, reduced hind paw swelling, lymphocyte infiltration and the destruction of cartilage tissue. Several reports have demonstrated the beneficial effects of *Lactobacillus* species in mouse models of arthritis however further research is needed to describe the mechanisms underlying its efficacy.

Animal studies support the thesis that *L. casei* strains down-regulate immune-system function (Vaghef-Mehrabany et al., 2018), which is beneficial in the case of RA and other inflammatory diseases. Results from a number of preclinical studies have demonstrated that various strains of *L. casei* can be effective in reducing arthritis score and decreasing serum inflammatory cytokines in RA (Kato et al., 1998; So et al., 2008; Amdekar et al., 2011, 2013; Pan et al., 2019). Although there are numerous studies reporting the efficacy of Lactic Acid Bacteria (LAB), their underlying mechanisms of action are still to be fully elucidated. Proposed mechanisms for *L. casei* strains include the improved proportion of Th2/Th1 cytokines, including the induction of Treg cells and down-regulation of Th1 cells. Once bound to TLR 2/6, *L. casei* bacteria, trigger various intracellular mechanisms, which will ultimately contribute to the maturation of FoxP3-CD4^+^ towards FoxP3 Treg cells (Vaghef-Mehrabany et al., 2018). Tregs cells are the main producers of IL-10, providing inhibitory effects on Th1 cells (Issazadeh-Navikas et al., 2012). Amdekar *et al.*, have also proposed a similar mechanism for *Lactobacillus* species (Amdekar et al., 2013). Therefore, the altered cytokine balance in favour of anti-inflammatory cytokines seems to be the main mechanism of action of probiotics in RA and should be the basis of future research. The role of antioxidant effects should be further elucidated.

#### Studies With the Adjuvant-Induced Arthritis Animal Model

Concerning the role of probiotic bacteria on disease progression, Pan et al. investigated the potential of administrating *L. casei* in the treatment of adjuvant-induced arthritis (AIA) and reported profound changes of microbial species in the gut as well as alterations in clinical signs during arthritis induction and progression phases, such as inhibition of joint swelling, lower arthritis scores, and prevention of bone destruction (Pan et al., 2019). Thirty days after prophylactic treatment with *L. casei*, a significant reduction in pro-inflammatory cytokines levels was observed. Moreover, an increased relative abundance of several *Lactobacillus* strains was also detected, such as *L. acidophilus, Lactobacillus hominis, L. reuteri, and Lactobacillus vaginalis*, suggesting that *L. casei* improves arthritis mainly through establishing the rebalance of the *Lactobacillus* strains (Pan et al., 2019). Some *Lactobacillus* strains have been reported to drive T cell differentiation from intraepithelial CD4^+^ T cells into immunoregulatory Treg. Also, their metabolic products, such as SCFAs, influence colonic Treg cell homeostasis (Smith et al., 2013; Cervantes-Barragan et al., 2017).

Rovensky et al. investigated the efficacy of *Escherichia coli O83* (Colinfant^®^) in the treatment of AIA (Rovenský et al., 2009). They studied the effect of Colinfant^®^ alone, of Colinfant^®^ in combination with MTX and MTX alone. They found a significant reduction in both inflammation and arthritis-associated alterations (reduction of hind paw swelling and arthrogram score) with MTX and with the combination of MTX and Colinfant^®^ (Rovenský et al., 2009). They also reported a more significant improvement of the arthritis score with combination treatment than with MTX alone. However, the use of Colifant^®^ alone had no impact on inflammatory markers (Rovenský et al., 2009).

Another study using an AIA model investigated whether *B. coagulans* and inulin, administered either isolated or in combination, influenced arthritis severity in rats (Abhari et al., 2016). A significant clinical improvement was observed in *B. coagulans* and/or inulin treated rats. This improvement included suppression of paw swelling and a decrease in pro-inflammatory parameters, such as fibrinogen and TNF-α (Abhari et al., 2016).

A study conducted by Achi et al. evaluated three strains of *Bifidobacteria*, namely *Bifidobacterium breve* NCIM 5671 (*B. breve* NCIM 5671), *Bifidobacterium longum* NCIM 5672, and *Bifidobacterium bifidum* NCIM 5697, to investigate their prophylactic effect in an AIA model (Achi et al., 2019). The results have demonstrated that *Bifidobacteria* can reduce the severity and progression of arthritis. However, *B. breve* NCIM 5671 had better antiarthritic effects in the rat model than the other bifidobacterial species studied, suggesting that the effect is strain-dependent, and these strains should be further explored for their putative positive impact on RA treatment (Achi et al., 2019). Table 1 summarizes the characteristics and main findings regarding probiotic effects on RA in preclinical studies.

**TABLE 1:** Probiotic effects on animal models of arthritis.

Animal models of CIA						
Author, year	Study objective	Animal model/administration timing	Probiotic strain	Administration dose	Evaluated parameters	Main findings
Kato et al. (1998)	To investigate the effects of *L. casei Shirota* on the development of CIA and immune responses	Male DBA 1 mice/after arthritis modeling	*L. casei* Shirota	PG1: 0.25 × 10^9^ CFU/day PG2: 0.5 × 10^9^ CFU/day PG3: 1 × 10^9^ CFU/day PG4:2 × 10^9^ CFU/day	Arthritis score; incidence of CIA; serum anti-CII antibodies; IFN-γ; IL-4	*L. casei Shirota* ↓ arthritis incidence in all groups; ↓ arthritis severity; ↓ CII-specific antibodies IgG2a and IgG2b; ↓ IFN-γ
So et al. (2008)	To investigate how *L. casei* suppresses the progression of CIA	Female lewis rats/before and after arthritis induction	*L. casei*	5 × 10^9^ CFU/dose	Paw swelling; arthritis score; CII-specific antibodies; CII-reactive pro-inflammatory molecules	*L.casei* ↓ hind paw swelling; ↓ lymphocyte infiltration; ↓ destruction of cartilage tissue; ↓ IL-1β; ↓ IL-2; ↓ IL-6; ↓ IL-12; ↓ IL-17; ↓ IFN-γ; ↓ TNF; ↓ COX-2; ↑ IL-10; ↓ serum CII-specific IgG2a and IgG2b; ↓ T cell proliferation (in both the pretreatment and acute phase treatment)
Amdekar et al. (2011)	To investigate the therapeutic efficacy of *L. casei* in a CIA model	Female wistar rats/after arthritis induction	*L.casei* ATCC 334	2 × 10^8^ CFU/ml	Arthritis score; serum cytokines; hind knee joint morphology	*L. casei* ↓ arthritis score; ↓ IL-6; ↓ TNF-α; ↓ infiltration of neutrophils in joint; ↓ bone erosion; ↓ pannus formation
Amdekar et al. (2013).	To evaluate antioxidant and anti-inflammatory potential of *L. casei* and *L. acidophilus* in a CIA model	Male wistar rats/after arthritis induction	*L.casei* ATCC 334 *L.acidophilus* ATCC314	PG1: 2 × 10^8^ CFU/ml (*L. casei*) PG2: 2 × 10^8^ CFU/ml (*L. acidophilus*)	Arthritis score; serum cytokines; oxidative stress markers (GSH, CAT, SOD, lipid peroxidation, GPx)	*L. casei* and L. acidophilus ↓ arthritis score; ↑ IL-4; ↑ IL-10; ↓ IL-6; ↓ TNF; ↓ IL-1β; ↓ IL-17; ↓ CAT; ↓ lipid peroxidation; ↑ GSH; ↑ GPx; ↑ SOD
Marietta et al. (2016)	To evaluate the effects of *P. histicola* for treating CIA	DQ8 mice/before and after arthritis induction	*P. histicola*	1 × 10^9^ live bacteria	Arthritis incidence; arthritis onset; arthritis severity; expression of TJ proteins; serum cytokines	*P. histicola* ↓ incidence of arthritis; ↓ severity of arthritis; ↓ IL-2; ↓ IL-17; ↓ TNF; ↑ IL-4; ↑ IL-10; ↓ anti-CII antibodies; ↓ gut permeability; ↑ ZO-1
Yamashita et al. (2017)	To evaluate the effect of *L. helveticus* on the development of CIA, antibody production and immune cells	Male DBA 1J mice/after arthritis induction	*L.helveticus* SBT2171	PG1:1.2 × 10^10^ CFU/g (oral administration) PG2: (Intraperitoneal inoculation)	Hind limb joint tissues; serum CII-specific antibodies; serum cytokines; total immune cells	*L. helveticus* oral administration ↓ joint swelling; ↓ body weight loss; ↓ serum CII-specific IgG and IgG1; *L. helveticus* intraperitoneal inoculation ↓ arthritis incidence; ↓ joint damage; ↓ serum IL-6; ↓ total B-cells; ↓ CD4^+^ T cells in the inguinal LNs
Fan et al. (2020)	To investigate the effects of *B. adolescentis* before and after arthritis induction on GM composition and immune responses	Female wistar rats/before and after arthritis induction	*B. adolescentis* cocktail including 5 strains HuNan2016-7-2 AHWH4-M1 FSDJN3Q1 M1DZ09M1 FSDJN12W5	5 × 10^9^ CFU/ml/day (per strain)	Ankle thickness; arthritis score; serum cytokines; serum anti-CII antibodies; tregs in MLNs; level of TJ proteins; GM composition; faecal SCFA	Preventive *B. adolescentis* performed better in ↓ ankle thickness; ↓ arthritis score; ↓TNF; ↑ Tregs in MLNs; ↑ SCFAs; ↑ mRNA level of ZO-1 and occludin; maintaining the gut microbial communities similar to the CG
**Animal models of AIA**						
Rovenský et al. (2009)	To evaluate the effect of *E. coli* O83 on AIA during basal treatment with MTX	Male lewis rats/after arthritis induction	*E. coli* O83	8 × 10^8^ bacteria/ml (1 ml/kg body mass)	Body mass; hind paw swelling; arthrogram score; serum albumin	E.*coli* O83 + MTX ↓ hind paw swelling; ↓ arthrogram score
Abhari et al. (2016)	To investigate the possible influence of *Bacillus coagulans* on immune responses and disease progression	Male wistar rats/before and after induction	*Bacillus coagulans*	10^9^ spores	Paw thickness; Fn; SAA; TNF-a; a1AGp	Pretreatment with *Bacillus coagulans* ↓ Fn; ↓ SAA; ↓ TNF
Achi et al. (2019)	To evaluate the prophylactic effect of B. breve NCIM 5671, B. longum NCIM 5672 and B. bifidum NCIM 5697 in a rat model of arthritis	Male wistar rats/before and after induction	*B. breve* NCIM 5671 *B. longum* NCIM 5672 *B. bifidum* NCIM 5697	10^8^–10^9^ cells/0.5 ml	Paw volume; bone mineral content; oxidative stress markers; antioxidant enzyme activity; serum cytokines; eicosanoids; expression of COX2	*Bifidobacterium* strains ↓ paw volume; ↓ PGE2; ↓ LTB4; ↓ LTC4; ↓ IL-1β; ↓ TNF; ↓ IL-6; ↓ MCP1; ↑ IL-4; ↑ IL-10; ↓ COX2 expression
Pan et al. (2019)	To evaluate the effect of *L. casei* for the treatment of arthritis	SD rats/after arthritis induction	*L. casei* ATCC334	2 × 10^8^ CFU/day	Hind paw volume; arthritis score; serum cytokines; GM composition	*L. casei* ↓ hind paw volume; ↓ arthritis score; ↓ IFN-γ; ↓ TNF; ↓ IL-1β; ↓ IL-17; ↓ IL-6; rebalance of the *lactobacillus* species; maintains the redox balance of oxidative stress

a1AGp, alpha-1-acid glycoprotein; AIA, adjuvant-induced arthritis; B, *bifidobacterium*; CAT, catalase; CFU, colony-forming units; CG, control group; CIA, collagen-induced arthritis; CII, type II collagen; COX-2, Cyclooxygenase-2; E, *escherichia*;; Fn, fibrinogen; GM, gut microbiota; GPx, glutathione peroxidase; GSH, glutathione; IFN, interferon; IL, interleukin; L, *lactobacillus*; LNs, lymph nodes; Tregs, regulatory T cells; MLNs, mesenteric lymph nodes; MTX, methotrexate; PG, probiotic group; SAA, serum amyloid A; SCFA, short chain fatty acids; SOD, superoxide dismutase; TJ, tight junction; TNF, tumour necrosis factor and ZO-1, zonula occludens-1.

### Summary of Evidence From Animal Models

Even after several decades of research, RA remains a complex disease of unknown etiology and without a cure (Choudhary et al., 2018). Animal models are widely used for testing potential new therapies for RA, and despite their recognized limitations, it is evident that these have provided valuable information regarding RA pathogenesis and the underlying mechanisms of disease. When considering all existing animal models of arthritis, the most commonly found question is which model is most predictive of therapeutic efficacy in human subjects with RA, as each model features a different mechanism driving disease expression (Hegen et al., 2008). Considering the problem to be investigated, the benefits of each model should be closely evaluated in order to make the most appropriate choice. Accordingly, for the identification and validation of drug targets, AIA and CIA models have great reproducibility and are the most commonly used models (Choudhary et al., 2018). The AIA model is characterized by acute inflammation and severe destruction, useful for the evaluation of the early structural consequences of arthritis and also for studies of pain pathways (Boissier and Bessis, 2017; Vidal et al., 2018). On the other hand, CIA has been an extremely popular model since its conception, once its underlying mechanisms involve numerous elements of the innate and adaptive immune systems, making it a useful model both for developing concepts to be extended to human subjects and for validating new treatment targets (Boissier and Bessis, 2017). The breach of tolerance and generation of auto antibodies towards self, are recognized as the most important characteristics of the CIA model, which makes it a very good *in vivo* model for RA studies (Asquith et al., 2009).

We have considered both prophylactic (when probiotic administration started before immunization or before arthritis onset) and therapeutic (when dosing with study probiotic started after clinical signs of disease) treatment regimens with probiotic bacteria in CIA and AIA models. A number of preclinical studies reported the beneficial effects of probiotics via multiple pathways, including restoring the dysbiosis of GM in a prophylactic way (Achi et al., 2019; Pan et al., 2019; Fan et al., 2020). A study conducted by Liu et al., has reported significant differences in the microbiome composition of CIA-susceptible and CIA-resistant mice (Liu et al., 2016). When transplanted to germ-free mice, the microbiome of the CIA-susceptible mice aggravated CIA disease severity, suggesting a relationship between GM composition and CIA susceptibility (Liu et al., 2016). These results showed that intestinal microbiota strongly affects the balance between pro- and anti-inflammatory immune responses in CIA. Although several studies reported differences in the microbiome composition of RA when compared to controls, little is known about the highly personalized microbiome dynamics during the induction, progression, and treatment of arthritis. The genus *Lactobacillus* is significantly more abundant prior to arthritis onset in CIA-susceptible mice than in CIA-resistant mice (Liu et al., 2016), However, results obtained by Pan et al., indicated that *L. casei* could influence the disordered microbiome and ameliorate arthritis via modulation of *Lactobacillus* strains (Pan et al., 2019). These findings highlighted the importance of monitoring changes in microbial communities during disease progression and provided powerful evidence to explain the evolution of the GM in RA. Of interest, Fan et al. reported the beneficial effects of early treatment in maintaining gut microbial communities (Fan et al., 2020).

There is no model fully reproducing a human rheumatic disease, which means that therapeutic interventions in animal models only provide partial information (Bessis et al., 2017). However, the development of novel treatment interventions for RA still relies on the careful analysis of studies in animal models combined with clinical observations.

### Experimental Evidence From Human Studies (Randomized Controlled Trials)

#### Randomized Controlled Trials With *Lactobacillus rhamnosus*

The effect of probiotic supplementation was studied in stable RA in a randomized controlled trial (RCT) evaluating the treatment with *Lactobacillus rhamnosus (L. rhamnosus)* GG versus placebo (Hatakka et al., 2003). Patients were not under treatment with DMARDs, but most of them were on stable medication with glucocorticoids (GC, 75% in the probiotic group and 62% in the placebo group) and non-steroidal anti-inflammatory drugs (NSAIDs, 75% in the probiotic group and 77% in the placebo group). In this study, the intervention group was given two capsules of *L. rhamnosus* (ATCC 53103) GG (Gefilus^®^, Valio Ltd.; ≥5 × 10^9^ colony-forming units (CFU) per capsule), twice a day, for 12 months, and the placebo group received identical capsules without the bacteria. There were no statistical differences in the clinical parameters, biochemical variables, and Health Assessment Questionnaire (HAQ) between groups. Inflammatory parameters were not significantly reduced. Interestingly, although there were no statistical differences in disease activity, more subjects in the intervention group reported subjective well-being when compared to the placebo group (Hatakka et al., 2003).

Supplementation with *L. rhamnosus* combined with *L. reuteri* was also studied as adjunctive therapy for patients with active RA (Pineda et al., 2011)*.* In this study, patients on stable medication (for at least one month) with DMARDs, steroids and/or NSAIDs were randomized to receive one capsule taken twice daily, for 3 months or placebo. The probiotic group received a supplement containing *L. rhamnosus* GR-1 and *L. reuteri* RC-14 (containing 2 × 10^9^ CFU/capsule). The placebo group received a capsule containing the same ingredients without the bacteria. A decrease in serum levels of IL-1a, IL-6, IL-10, IL-12p70, and TNF was reported, but placebo caused a significantly greater decline in the production of IL-6, IL-12p70, and TNF, as well as IL-15, IL-17. Finally, although there was a significant improvement in the HAQ score in the probiotic group, no between-group differences were found (Pineda et al., 2011).

#### Randomized Controlled Trials With *Lactobacillus casei*

A different strain of *Lactobacillus, L. casei,* was also studied for its potential benefits in RA. In the study conducted by Vaghef-Mehrabany et al., patients with inactive to moderate RA (i.e., a disease activity score (DAS28) of <5.1) who were following a stable medication regimen for at least three months were included; current medication for most patients included DMARDs and GCs, but not NSAIDs or biologics. The probiotic group received a daily capsule of *L. casei* 01 (>10^8^ CFU/capsule) for eight weeks (Vaghef-Mehrabany et al., 2014). The placebo group received capsules containing only maltodextrin (the excipient used in the probiotic capsules). The probiotic supplementation significantly decreased three of the assessed serum pro-inflammatory cytokines (TNF, IL-6, and IL-12). Moreover, a significant increase in serum levels of IL-10 was observed in the probiotic group. In this study, the pain visual analogue scale (VAS) score decreased, compared with baseline, by 43.96% in the probiotic group and by 5.99% in the placebo group at the end of the study (Vaghef-Mehrabany et al., 2014). Regarding the effects of probiotic supplementation with *L. casei* on oxidative stress, Vaghef-Mehrabany et al. conducted a secondary analysis and concluded that this intervention had no significant effect on the oxidative status of patients with RA compared to placebo (Vaghef-Mehrabany et al., 2016). Additionally, the same authors found that this intervention with *L. casei* 01 also significantly decreased serum high sensitivity C-reactive protein (hs-CRP), global health score (assessed by VAS), DAS-28, as well as tender and swollen joint counts (Alipour et al., 2014). Regarding disease activity, DAS-28 (mean ± standard deviation) decreased from 2.56 ± 1.01 at baseline to 2.07 ± 0.82 at the end of the study in the probiotic group, while a much smaller improvement in DAS-28 was observed in the placebo group (2.31 ± 0.90 at baseline to 2.23 ± 0.86 at the end of the study) (Alipour et al., 2014).

Zamani et al. tested *L. casei* combined with other strains (Zamani et al., 2016). In this RCT, the intervention group received, in addition to their conventional medications (DMARDs and GCs), a daily capsule containing *L. casei* (2 × 10^9^ CFU/g), *L. acidophilus* (2 × 10^9^ CFU/g) and *B. bifidum* (2 × 10^9^ CFU/g) for eight weeks, and the placebo group took capsules filled with cellulose for the same amount of time. Although this trial was conducted in patients with moderate to severe disease activity (DAS-28 > 3.2), contrarily to the previous studies, probiotic supplementation also resulted in improved DAS-28. In the probiotic group, DAS-28 (mean ± standard deviation) decreased from 4.0 ± 0.7 at baseline to 3.7 ± 0.7 at the end of the trial, while the decrease in the placebo group was only from 4.1 ± 0.7 at baseline to 4.0 ± 0.7 at the end of the trial. The authors also found a significant decrease in serum hs-CRP concentrations and other parameters not previously studied, such as serum insulin levels (Zamani et al., 2016). In line with Vaghef-Mehrabany et al., this intervention did not influence biomarkers of oxidative stress compared with the placebo among patients with RA (Zamani et al., 2016).

Lastly, *L. casei* was also tested combined with other strains in the study conducted by Cannarella et al. (Cannarella et al., 2021). In this trial, RA patients in the probiotic group took a daily sachet for 60 days containing 10^9^ CFU/g of each of the following strains: *L. casei* LC-11, *L. acidophilus* LA-14, *Lactococcus lactis* LL-23, *B. lactis* BL-04 and *B. bifidum* BB-06, and the placebo group took maltodextrin daily for the same amount of time. Similarly to the previous studies, the usual medication was maintained in both groups during the experiment. The probiotic group showed a significant reduction in white blood cell counts, TNF and IL-6 plasma levels, but this intervention did not alter DAS-28 (median of 3.83 at baseline vs a median of 3.88 at the end of the study in the placebo group; median of 3.20 at baseline vs a median of 3.18 at the end of the study in the probiotic group). Moreover, no differences were found in the IL-10, adiponectin, CRP and erythrocyte sedimentation rate (ESR) between groups. The authors also assessed parameters that were not reported in the previous studies, such as oxidative/nitrosative profile and antioxidant defences. They found that probiotic supplementation improved the oxidative/nitrosative profile and increased the antioxidant defences in patients with RA. In this regard, the probiotic group showed lower nitric oxide metabolites, and higher sulfhydryl group and a total radical-trapping antioxidant parameter compared to the placebo group (Cannarella et al., 2021).

#### Randomized Controlled Trials With *Bacillus coagulans*

An RCT with *B. coagulans* GBI-30, 6,086® (GanedenBC30^®^), also used in combination with DMARDs, was conducted by Mandel et al. in RA patients. In this trial, besides the probiotic itself, the preparation included green tea extract, methylsulfonylmethane, vitamins and minerals, and the placebo contained microcrystalline cellulose. There was a statistically significant improvement in the pain scale compared to placebo (Mandel et al., 2010). Besides, the probiotic supplementation resulted in a reduction of CRP and greater improvement in patient-global assessment, self-assessed disability, the ability to walk two miles and participation in daily activities compared to placebo (Mandel et al., 2010). The authors did not report DAS-28 but suggested that *B. coagulans* GBI-30, 6,086® supplementation may be an effective adjunct therapy for the relief of RA symptoms. Trials assessing disease activity are warranted.

### Summary of Evidence From Randomized Controlled Trials

**FIGURE 3:**
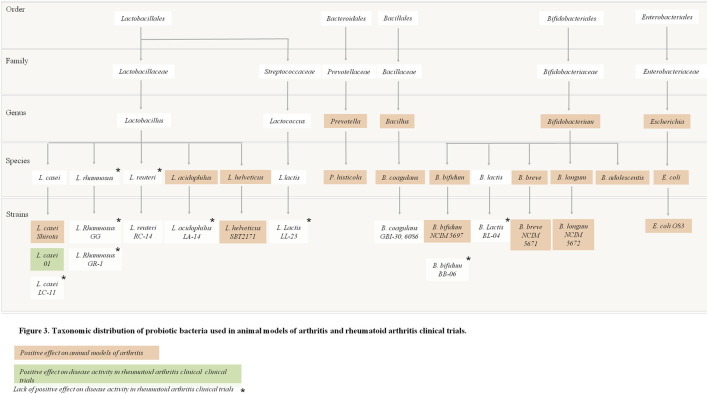
Taxonomic distribution of probiotic bacteria used in animal models of arthritis and rheumatoid arthritis trials.

Overall, there is some evidence from human RCTs that probiotic supplementation can improve disease activity and the inflammatory status of RA patients when used in addition to the patient’s conventional medications. Additionally, the recently published RCT by Cannarella et al. reported that probiotic supplementation could also increase the antioxidant defences and improve oxidative/nitrosative profile in RA patients (Cannarella et al., 2021). Figure 3 summarizes the experimental evidence for the use of probiotic bacteria in RA according to taxonomic distribution.

Several different strains have been tested for their benefit in RA patients, as single species or in mixed culture, in different dosages, which is a limitation that could explain conflicting results. Selecting the most appropriate strain for administration to RA patients is of major importance, and this should be sought in future research projects. As of now, probiotic supplementation with *L. casei* seems to be the strongest candidate to be used as adjuvant therapy for RA patients, and current evidence suggests a minimum of 10^8^ CFU/capsule per day to obtain significant results. Supporting this, a systematic review and meta-analysis investigating the effectiveness of probiotic supplementation in RA underlined that the trials in which a significant reduction of CRP was achieved used the same probiotic strain, *L. casei* (Rudbane et al., 2018). Nevertheless, strain choice is only one of the various variables that are likely to influence the outcomes of probiotic studies, because dosage, study duration, frequency of administration, baseline disease activity (Marco and Tachon, 2013) and concomitant pharmacological treatment are also of major importance. Table 2 details the characteristics and main findings of the included papers regarding probiotic effects on RA.

**TABLE 2:** Probiotic effects on Rheumatoid Arthritis in clinical trials.

	Author/ year	Sample size/ study duration/ RA severity			Probiotic strain	Administration dose	Evaluated Parameters	Main findings
RCT with L. rhamnosus	(Hatakka et al., 2003)		*n* = 21/12 months/mild disease	L. rhamnosus GG		≥5 × 10^9^ CFU/capsule (4 caps/day)	HAQ; TJC and SJC; ESR; CRP; IL-1β; IL-6; IL-10; IL-12; TNF	No statistical differences were observed; ↑ number of subjects reporting subjective well being in the probiotic group
	(Pineda et al., 2011)		*n* = 29/3 months/severity not described	L. rhamnosus GR-1L. reuteri RC-14		2 × 10^9^ CFU/capsule (2 caps/day)	TJC and SJC; ESR; CRP; TNF; IL-1 α; IL-1β; IL-6; IL-10; IL-12; GH VAS; pain VAS; HAQ	No statistical differences were observed between groups
RCT with L.casei	(Vaghef-Mehrabany et al., 2014)		*n* = 46/8weeks/mild to moderate disease	L. casei 01		≥10^8^ CFU/capsule (1 caps/day)	IL-1β; IL-6; IL-10; IL-12;TNF	↓ IL-6; ↓ IL-12; ↓ TNF;↑ IL-10
	(Alipour et al., 2014)		*n* = 46/8 weeks/mild to moderate disease	L. casei 01		≥10^8^ CFU/capsule (1 caps/day)	DAS28; TJC and SJC; GH score; hs-CRP	↓ DAS28;↓ hs-CRP; ↓ GH score; ↓ TJC and SJC
	(Vaghef-Mehrabany et al., 2016)		*n* = 46/8 weeks/mild to moderate disease	L. casei 01		≥10^8^ CFU/capsule (1 caps/day)	MDA; SOD; GPx; CAT; TAC	No significant effect was observed on the oxidative status
	(Zamani et al., 2016)		*n* = 60/8 weeks/moderate to severe disease	L. acidophilusL. caseiB. bifidum		2x10^9^ CFU/g of each strain (1 caps/day)	DAS28; TJC and SJC; VAS of pain; hs-CRP; serum insulin; HOMA-B; HOMA-IR; lipid profile; NO; TAC; GSH; MDA	↓ DAS28;↓ serum insulin; ↓ HOMA-B;↓ hs-PCR
	(Cannarella et al., 2021)		*n* = 47/60 days/severity not described	L. casei LC-11L. acidophilus LA-14Lactococcus lactis LL-23B. Lactis BL-04B. Bifidum BB-06		10^9^ CFU/sachet of each strain (1 sachet/day)	DAS28; GH VAS; TJC and SJC; WBC; ESR; hs-CRP; TNF; IL-6; IL-10; adiponectin; LOOH; PC; NO; SH	↓ WBC; ↓ TNF;↓ IL-6; ↓ NOx;↑ Total antioxidant capacity (TRAP); ↑ SH
RCT with *Bacillus coagulans*	(Mandel et al., 2010)		*n* = 45/60 days/severity not described	Bacillus Coagulans GBI-30, 6086		2x10^9^ CFU/day	HAQ-DI; TJC and SJC; ERS; CRP; pain score; global assessment	↓ pain score and CRP

B, *bifidobacterium*; CAT, catalase; CRP, C-reactive protein; DAS28, disease activity score 28 joint count; ESR, erythrocyte sedimentation rate; GH, global health; GSH, glutathione; GPx, glutathione peroxidase; HAQ, Health Assessment Questionnaire; HAQ-DI, Stanford Health Assessment Questionnaire Disability Index; HOMA-B, homeostatic model assessment-B cell function; HOMA-IR, homeostasis model of assessment-estimated insulin resistance; hs-CRP, serum high sensitivity C-reactive protein; IL, interleukin; L, *lactobacillus*; LOOH, lipid hydroperoxide; MDA, malondialdehyde; NO, nitric oxide; NOx, nitric oxid metabolites; PC, protein carbonyl; RCT, randomized clinical trial; RA, rheumatoid arthritis; SH, sulfhydryl groups; SJC, swollen joint counts; SOD, superoxide dismutase; TAC, total antioxidant capacity; TJC, tender joint counts; TNF, tumour necrosis factor; TRAP, total radical-trapping antioxidant parameter; VAS, visual analogue scale; WBC, white blood cell counts.

## Fermented Foods as a Possible Alternative to Probiotic Supplements

Fermented foods and beverages are defined as foods made through desired microbial growth and enzymatic conversions of food components (Marco et al., 2021). *Yoghurt, Kefir, Miso, Natto, Tempeh* and most *Kombuchas* are some of the available fermented foods that contain live microorganisms (Marco et al., 2021). Historically, many foods have undergone fermentation as a food preservation technique (Paul Ross et al., 2002; Dimidi et al., 2019) since the generation of antimicrobial metabolites like organic acids, ethanol and bacteriocins reduce the risk of contamination with pathogenic microorganisms (Dimidi et al., 2019). Nowadays, fermented foods and beverages are more popular than ever before (Bell et al., 2018). This emerging interest in such foods could be explained by their health-promoting potential (Marco et al., 2017; Melini et al., 2019).

Although the current body of evidence regarding the impact of fermented foods on health and disease remains insufficient (Bell et al., 2018; Dimidi et al., 2019), it is increasingly understood that some fermented foods promote human health in ways not directly attributable to the starting food materials (Marco et al., 2017). These foods could benefit health through the nutritive alteration of the ingredients, modulation of the immune system, or by influencing GM composition and activity (Marco et al., 2021). Most likely, several of these mechanisms are related and contribute to the effects of each other.

These foods contain various microbes with health-promoting properties, and GM has been suggested to be the mediator between fermented food consumption and these health outcomes. However, it is important to acknowledge that changes in the bacterial composition of diet do not necessarily translate into GM functional changes (Stiemsma et al., 2020). That being said, there is evidence from dietary interventions in humans suggesting that foodborne microbes can transiently colonize gut (David et al., 2014). Food ingested bacteria are capable of transient integration into GM, despite the resistance of resident communities to colonization by ingested bacteria (Derrien and van Hylckama Vlieg, 2015). Although these microorganisms are unlikely to maintain long-term residence in the intestine, it has been suggested that short-term colonization could be sufficient to synthesize bioactive compounds, inhibit intestinal pathogens and mediate epithelial modulatory effects (Marco et al., 2021). Nevertheless, to fully understand the long-term effects of the consumption of fermented foods, it is crucial that future studies have longer intervention periods and evaluate the GM composition, not only before and immediately after the intervention, but also some time after the cessation of the regime (e.g., several weeks). *Bifidobacterium* and lactic acid bacteria, including *Lactobacillus*, which were studied as probiotic supplements in RA patients, are some of the microorganisms present in many fermented foods (Tamang et al., 2016).

Even though fermented foods can be classified as probiotics if they meet the required criteria, it is important to clarify that fermented foods are not equivalent to probiotic foods since many fermented foods do not have evidence of a demonstrated health benefit conferred by well-defined and characterized live microorganisms (Hill et al., 2014; Marco et al., 2021). The effects of the microorganisms are strain specific and most likely dose-dependent. This is one of the main limitations regarding the use of fermented foods as an alternative to probiotic supplementation. Additionally, some foods and beverages produced by fermentation do not contain live microorganisms, such as bread, beer, wine and distilled alcoholic beverages, due to their inactivation by heat or physical removal by filtration or other means (Rezac et al., 2018). As previously discussed, fermented foods could lead to beneficial outcomes by various mechanisms besides providing living microorganisms to the gastrointestinal tract. Hence, these foods could carry a positive functional role even in the absence of live microorganisms in the finished product, considering that microbes are capable of modifying food constituents, may produce vitamins or other bioactive molecules and inactivate anti-nutritional factors (Marco et al., 2017; Rezac et al., 2018). Since several other components in the food matrix may positively influence health and it is possible that fermented foods carry additional benefits when compared with probiotic supplementation by itself.

Considering all these findings, fermented foods can be probiotic carriers and may be a promising alternative to probiotic supplementation for RA patients. Fermented foods may change the amounts and types of beneficial bacteria that live in human gut (Stiemsma et al., 2020) and, considering the emerging evidence linking dysbiosis with autoimmunity mechanisms (Horta-Baas et al., 2017), this could be particularly interesting for RA patients. Moreover, there is evidence of the beneficial effect of fermented foods in reducing inflammatory biomarkers in studies conducted in healthy individuals (Burton et al., 2017; Pei et al., 2017). Of interest to the subject discussed in this paper, fermented foods have also been studied in other inflammatory conditions and, although more research is needed, results suggested that these foods can exert beneficial effects in conditions characterized by chronic inflammation. For instance, an RCT conducted in patients with inflammatory bowel disease found that *kefir* consumption significantly decreased ESR and CRP serum levels in patients with Crohn’s disease and concluded that this intervention may improve both symptoms and quality of life of these patients (Yılmaz et al., 2019). Furthermore, in patients with type 2 diabetes, the consumption of a probiotic yogurt containing 3.7 × 10^6^ CFU/g of both *L. acidophilus* (La5) and *B. lactic* (Bb12), significantly decreased TNF levels (Mohamadshahi et al., 2014). A significant decrease in TNF levels, as a result of yogurt consumption, was also found in a RCT promoted by Chen et al., conducted in obese women with nonalcoholic fatty liver disease and metabolic syndrome (Chen et al., 2019). In this study conventional yogurt was used and a significant decrease in serum lipopolysaccharide (LPS), a biomarker of gut permeability, was also found as well as changes in GM composition, namely a decrease in the abundance of the *Firmicutes* phylum and the taxa within it (Chen et al., 2019). In line with this, a recent systematic review and meta-analysis of RCTs regarding fermented foods and inflammation reported that fermented foods might have beneficial effects in subjects with an inflammatory disease background (SaeidiFard et al., 2020), as is the case of RA patients. Lastly, dietary interventions are among the nonpharmacological therapies proposed to minimize the consequences of the disease in patients with established RA (Küçükdeveci, 2019). Finally, it has already been suggested that fermented foods may be an appealing complement to a whole-dietary pattern, such as the Mediterranean Diet, since both fermented foods and the Mediterranean Diet have similar anti-inflammatory pathways and may potentiate each other, resulting in a promising combination for RA patients (Dourado et al., 2020). Altogether, this evidence highlights the need for well conducted intervention studies with fermented foods in RA patients.

## Conclusion

The link between gut dysbiosis and RA has expanded the interest in investigating the modulation of the GM as a possible adjuvant therapy for disease prevention and treatment. The increasing evidence reporting the positive effects of probiotic bacteria in animal models of arthritis has been leveraging the desire to transfer these benefits into clinical practice. However, only a small number of studies addressed the role of probiotics in the management of RA on human subjects and, to the best of our knowledge, no human trial has investigated the role of probiotics in a preventive approach. Research in this field is still in need of high-quality studies with larger sample sizes and longer treatment durations to ascertain the exact benefit of this promising treatment for RA patients.

Probiotics supplementation in RA seems to have no clinically significant adverse effects, but further research is needed to get a solid basis concerning the most appropriate strains for RA patients. As of now, *L. casei* seems to be the strongest candidate, and its potential effect on GM and immune system could be further explored to achieve new insights on this promising therapy for RA patients.

Moreover, fermented foods may be a possible alternative to probiotic supplementation, as some of these foods and beverages are known to be probiotic carriers with potentially similar health benefits. As the current body of evidence investigating the impact of fermented foods on health and disease remains insufficient, its proposed benefits on the human GM should warrant future research consideration.
